# Life’s Tenure

**Published:** 1872-02

**Authors:** 


### LIFE'S TENURE

Is very short and very slight sometimes, making the Scripture announcement literally true, " In the midst of life we are in death." The sword of Damocles is forever hanging over humanity, from the cradle to the grave, proving the truth of the words of Isaac Watts, of immortal and glorious memory : —

> " Our life contains a thousand springs,
> And dies if one be gone ;
> Strange that a harp of thousand strings
> Should keep in tune so long."

In wringing out a dress at Oil City, lately, a young woman had a needle run through the palm of her hand, of which she died within five minutes. It is not at all likely that a similar incident has occurred since the foundation of the world.

A little daughter of Dr. Moore, of Poughkeepsie, was playing on the floor, and an open pen-knife fell on the side of her neck, point foremost, severing the carotid artery ; she died in eight minutes.

Recently, a young woman was hanging clothes on a line to dry, and a bee stung her on the forehead ; she died before she got into the house. Incidents like these press upon our attention, most forcibly, the truth of that Scripture, and its wisdom, too, which counsels us all, most lovingly, "Be ye also ready, for in such an hour as ye think not, the Son of man cometh."

